# The Role of *relA* and *spoT* in *Yersinia pestis* KIM5^+^ Pathogenicity

**DOI:** 10.1371/journal.pone.0006720

**Published:** 2009-08-24

**Authors:** Wei Sun, Kenneth L. Roland, Christine G. Branger, Xiaoying Kuang, Roy Curtiss

**Affiliations:** Center for Infectious Disease and Vaccinology, The Biodesign Institute and School of Life Sciences, Arizona State University, Tempe, Arizona, United States of America; Cairo University, Egypt

## Abstract

The ppGpp molecule is part of a highly conserved regulatory system for mediating the growth response to various environmental conditions. This mechanism may represent a common strategy whereby pathogens such as *Yersinia pestis*, the causative agent of plague, regulate the virulence gene programs required for invasion, survival and persistence within host cells to match the capacity for growth. The products of the *relA* and *spoT* genes carry out ppGpp synthesis. To investigate the role of ppGpp on growth, protein synthesis, gene expression and virulence, we constructed a *ΔrelA ΔspoT Y. pestis* mutant. The mutant was no longer able to synthesize ppGpp in response to amino acid or carbon starvation, as expected. We also found that it exhibited several novel phenotypes, including a reduced growth rate and autoaggregation at 26°C. In addition, there was a reduction in the level of secretion of key virulence proteins and the mutant was>1,000-fold less virulent than its wild-type parent strain. Mice vaccinated subcutaneously (s.c.) with 2.5×10^4^ CFU of the Δ*relA* Δ*spoT* mutant developed high anti-*Y. pestis* serum IgG titers, were completely protected against s.c. challenge with 1.5×10^5^ CFU of virulent *Y. pestis* and partially protected (60% survival) against pulmonary challenge with 2.0×10^4^ CFU of virulent *Y. pestis*. Our results indicate that ppGpp represents an important virulence determinant in *Y. pestis* and the Δ*relA* Δ*spoT* mutant strain is a promising vaccine candidate to provide protection against plague.

## Introduction

Plague remains one of the most feared infectious diseases in humans. The etiological agent of the disease, *Yersinia pestis*, is disseminated by fleas and infects both humans and rodents. *Y. pestis* rapidly invades from the infection site into the lymphatic system and circulation, to produce the systemic and often fatal disease [1]. Globally about 2000 cases of plague are reported to the World Health Organization each year [2]. Most of these cases are the bubonic form of the disease, usually a consequence of the transmission of bacteria to humans via bites from fleas that have previously fed on infected rodents although contact with domestic cats that have been exposed to *Y. pestis* is another important transmission mode because of the higher than average incidence of pneumonic plague that occurs in these cases [1]. More rarely, cases of pneumonic plague are reported that are characterized by a short incubation period of 2 to 3 days and a high rate of mortality, even if treated. Pneumonic plague can be transmitted person to person or animal to person via the inhalation of contaminated air droplets [1]. Pneumonic plague is the most likely form to be encountered if *Y. pestis* is used as a biological weapon [3].

*Y. pestis* overwhelms its mammalian host during systemic growth by evading phagocytosis and by inhibiting the inflammatory response [4]. These properties are associated with a 70-kb plasmid, termed pCD1, which codes for a virulence-associated type III secretion system (T3SS) [5]. The analogous 70-kb pYV (*Yersinia* virulence) plasmid is found in *Yersinia pseudotuberculosis* and *Yersinia enterocolitica* which primarily cause gastrointestinal disease. In addition, *Y. pestis* has two plasmids that are absent in other yersiniae: the 100-kb plasmid pMT1 proposed to contribute to the survival of the bacteria in the flea [6], and the 9.5-kb plasmid pPCP1, which is responsible for the invasive character of plague in the mammalian host [7]. After subcutaneous (s.c.) administration of *Y. pestis* in mice, the pPCP1 plasmid potentiates the spread of bacteria into the circulation [7]. It was recently demonstrated that pPCP1 also enhances invasion of *Y. pestis* into human epithelial cells [8].

The stringent response is a generalized adaptive response to nutritional deprivation and environmental stress. The production of a specific nucleotide, guanosine-5′,3′-(bis)pyrophosphate (ppGpp), is the primary signaling and initiating event in the stringent response. In *Escherichia coli* and *Salmonella enterica* serovar Typhimurium, the *relA* gene encodes an enzyme with guanosine 3′,5′ bis(diphosphate) synthetase activity, which carries out ppGpp synthesis in response to a nutritional imbalance caused by amino acid starvation leading to increased synthesis of ppGpp and a reduction in stable RNA synthesis. This response to amino acid limitation is absent in Δ*relA* strains [9]. Basal levels of ppGpp synthesized in the absence of *relA* activity during balanced growth is regulated by *spoT*, which encodes an enzyme with both guanosine 3′, 5′-bis(diphosphate) 3′-pyrophohydrolase and guanosine 3′,5′ bis(diphosphate) synthetase activity [10]. The *spoT* gene modulates ppGpp levels in response to a number of nutritional factors including carbon starvation [10]. In Gram-positive bacteria, a single RelA/SpoT protein is responsible for both functions [11]. The *relA* and *spoT* genes play an important role in the virulence of a number of pathogenic bacteria, including *Mycobacterium tuberculosis* [12], *Listeria monocytogenes* [13], *Legionella pneumophila* [14], [15], *Vibrio cholerae* [16], and *Pseudomonas aeruginosa* [17]. A recent study indicated that a Δ*relA* Δ*spoT S.* Typhimurium is effectively noninvasive for epithelial cells *in vitro* and is attenuated in BALB/c mice [18], [19]. These genes were shown to play a crucial role in the regulation of genes in *Salmonella* pathogenicity islands 1 and 2 (SPI1, SPI2) and the *spv* virulence plasmid genes. Taken together, these results suggest that ppGpp may play a universal role in bacterial virulence gene expression.

The ppGpp molecule is part of a highly conserved regulatory system for mediating the growth response to various environmental conditions. This mechanism may represent a common strategy whereby facultative intracellular pathogens regulate the virulence gene programs required for invasion, survival and persistence within host cells to match the capacity for growth. However, the role of the ppGpp in *Y. pestis* physiology and virulence has not been investigated.

The goal of our study was to determine what role *relA* and *spoT* play in *Y. pestis* physiology and virulence by constructing *ΔrelA* and *ΔrelA ΔspoT* mutants and characterizing them for both in vitro and in vivo characteristics. We examined the effect of these mutations on transcription and protein levels at 26°C (flea temperature) and at 37°C (human temperature) and the effect on host colonization, immune responses and virulence. We also evaluated the double mutant for its capacity to induce protective immunity. Our results showed that the *relA spoT* mutant was attenuated for virulence and induced protective immunity by s.c. vaccination against bubonic and pneumonic plague.

## Materials and Methods

### Bacterial strains, culture conditions and plasmids

All bacterial strains and plasmids used in this study are listed in Table 1. All strains were stored at −70°C in phosphate-buffered glycerol. *Y. pestis* cells were grown routinely at 28°C on Congo red agar from glycerol stocks and then grown in heart infusion broth (HIB) or on tryptose-blood agar base (TBA) [20]. The chemically defined medium PMH2 was used routinely [21]. All *E. coli* strains were grown routinely at 37°C in LB broth [22] or LB solidified with 1.2% Bacto Agar (Difco).

**Table 1:** Bacterial strains and plasmids used in this study.

Strains	Relevant genotype or Annotation	Source or derivation
*E.coli* TOP10	F^−^ *mcrA* Δ(*mrr*-*hsdRMS*-*mcrBC*) φ80*lac*ZΔ*M15* Δ*lacX74 recA1 araD139* Δ (*ara*-*leu*)*7697 galU galK rpsL endA1 nupG*	Invitrogen
*Y. pestis* KIM6^+^	Pgm^+^, pMT1, pPCP1, cured of pCD1	[21]
*Y. pestis* KIM5^+^	*Y. pestis* KIM6^+^ pCD1Ap	[21]
χ10003	Δ*relA233 Y. pestis* KIM6^+^	[24]
χ10004	Δ*relA233* Δ*spoT85 Y. pestis* KIM6^+^	[24]
χ10019	Δ*relA233* Δ*spoT85* Δ*lacZ516*::TT *araC* P_BAD_ *spoT Y. pestis* KIM6^+^	This study
χ10021	*spoT412*:: 3×Flag-*Kan Y. pestis* KIM6^+^	This study
χ10022	Δ*relA233* Δ*spoT85* Δ*lacZ*ΩTT *araC* P_BAD_ *spoT413*:: 3×Flag-*Kan Y. pestis* KIM6^+^	This study
χ10023	Δ*pla-525 Y. pestis* KIM6^+^	This study
χ10024	Δ*relA233* Δ*pla-525 Y. pestis* KIM6^+^	This study
χ10025	Δ*relA233* Δ*spoT85* Δ*pla-525 Y. pestis* KIM6^+^	This study
χ10026	Δ*relA233* Δ*spoT85* Δ*pla-525* Δ*lacZ516*::TT *araC* P_BAD_ *spoT Y. pestis* KIM6^+^	This study
χ10003(pCD1Ap)	Δ*relA233 Y. pestis* KIM6^+^ pCD1Ap	This study
χ10004(pCD1Ap)	Δ*relA233* Δ*spoT85 Y. pestis* KIM6^+^ pCD1Ap	This study
χ10019(pCD1Ap)	Δ*relA233* Δ*spoT85* Δ*lacZ*::TT *araC* P_BAD_ *spoT Y. pestis* KIM6^+^ pCD1Ap	This study
χ10023(pCD1Ap)	Δ*pla-525 Y. pestis* KIM6^+^ pCD1Ap	This study
χ10024(pCD1Ap)	Δ*relA233* Δ*pla-525 Y. pestis* KIM6^+^ pCD1Ap	This study
χ10025(pCD1Ap)	Δ*relA233* Δ*spoT85* Δ*pla-525 Y. pestis* KIM6^+^ pCD1Ap	This study
χ10026(pCD1Ap)	Δ*relA233* Δ*spoT85* Δ*pla-525* Δ*lacZ516*::TT *araC* P_BAD_ *spoT Y. pestis* KIM6^+^ pCD1Ap	This study
Plasmids		Source
pUC18	For cloning and sequencing	Invitrogen
pCD1Ap	70.5-kb pCD1 with *bla* cassette inserted into '*yadA*; 71.7-kb Lcr^+^ Ap^r^	[21]
pCP20	Ap^r^ Cm^r^, FLP recombinase expression	[26]
pKD3	Ap^r^ Cm^r^, *cat* cassette template	[26]
pKD46	Ap^r^, λ Red recombinase expression	[26]
pYA3700	TT *araC* P_BAD_ cassette plasmid, Ap^r^	[85]
pSUB11	Kn^r^, 3xFlag-tagged	[25]
pYA4373	The *cat*-*sacB* cassette in the *Pst*I and *Sac*I sites of pUC18.	pUC18
pYA4573	The *lacZ-*U (upstream gene sequence of *lacZ*), and *lacZ-*D (downstream gene sequence of *lacZ*) fragment were cloned into the *Sph*I/*Pst*I sites and *Sac*I/*Eco*RI sites of pYA3700 respectively.	pYA3700
pYA4574	The *spoT* gene with new SD sequence was cloned into the *Xho*I and *Sac*I sites of pYA4573.	pYA4573
pYA4575	The *cat*-*sacB* cassette from pYA4373 was ligated into *Pst*I site of pYA4574.	pYA4574
pYA4642	The C-terminal *spoT* gene fragment (510 bp) was cloned into *Hind*III and *Bam*HI sites of pUC18.	pUC18
pYA4643	The *spoU'* gene fragment (downstream sequence of *spoT*) was cloned into *Sac*I and *Eco*RI sites of pYA4642.	pYA4642
pYA4644	The *lacZ*-D gene fragment (downstream sequence of *lacZ*) was cloned into *Sac*I and *Eco*RI sites of pYA4642.	pYA4642
pYA4645	The 3×Flag::*kan* gene fragment was cloned into *Sac*I and *Bam*HI sites of pYA4643.	pYA4643
pYA4646	The 3×Flag::*kan* gene fragment was cloned into *Sac*I and *Bam*HI sites of pYA4644.	pYA4644
pYA4647	The *pla*-U fragment (upstream sequence of *pla*) was cloned into the *Eco*RI and *Pst*I sites of pUC18.	pUC18
pYA4648	The *pla*-D fragment (downstream sequence of *pla*) was cloned into the *Sph*I and *Pst*I sites of pYA4647.	pYA4647
pYA4649	The *cat* cassette (including Flp recombination site) was cloned into the *Pst*I site of pYA4648.	pYA4648

### Plasmid construction

All primers used in this paper are listed in Table S1. The original source for the tightly regulated *araC* P_BAD_ in pYA3700 was *E. coli* K-12 strain χ289 [23]. For construction of the P_BAD_ *spoT* insertion/deletion into *lacZ*, primer sets of LacZ1/LacZ2 and LacZ3/LacZ4 were used for amplifying *lacZ-*U (upstream gene sequence of *lacZ*), and *lacZ-*D (downstream gene sequence of *lacZ*) fragment, respectively. The *lacZ*-U and *lacZ*-D fragments were cloned into the *Sph*I/*Pst*I sites and *Sac*I/*Eco*RI sites of pYA3700 to form pYA4573. The *spoT* gene fragment was amplified using SpoT-1 and SpoT-2 primers. The primer SpoT-1 containing the new SD sequence is shown Table S1. The *spoT* fragment was cloned into pYA4573 to construct pYA4574. Plasmid pYA4574 was digested with *Pst*I, blunt ended with T4 DNA polymerase and dephosphorylated with Shrimp Alkaline phosphatase (Promega). The *cat-sacB* fragment was cut from pYA4373 using *Pst*I and *Sac*I restriction endonucleases and blunted by T4 DNA polymerase. Then, the *cat-sacB* fragment was ligated into *Pst*I site of pYA4574 to form plasmid pYA4575.

To construct a *spoT*-3x-*flag-kan* fusion, a C-terminal *spoT* gene fragment (510 bp) was amplified using SpoTC-1 and SpoTC-2 primers and cloned into *Hind*III and *Bam*HI sites of pUC18 to construct pYA4642. The *spoU'* gene fragment (sequence downstream of *spoT*) and *lacZ*-D gene fragment (sequence downstream of *lacZ*) were amplified from genomic DNA using SpoTD-1/SpoTD-2 and LacZ3/LacZ4 primers, respectively. The *spoU'* and *lacZ*-D fragment were cloned into *Sac*I and *Eco*RI sites of pYA4642 to form pYA4643 and pYA4644, respectively. Then the 3×*flag*-*kan* gene fragment amplified from pYA4045 was cloned into *Sac*I and *Bam*HI sites of pYA4643 and pYA4644 to construct pYA4645 and pYA4646.

To delete the *pla* gene from plasmid pPCP1, plasmids pYA4647, pYA4648, and pYA4649 were constructed. The *pla*-U fragment was amplified from total DNA of *Y. pestis* KIM6^+^ using Pla1 and Pla2 primers and cloned into the *Eco*RI and *Pst*I sites of pUC18 to form pYA4647. The *pla*-D fragment was amplified using Pla3 and Pla4 primers. The *pla*-D fragment was cloned into pYA4647 to construct pYA4648. The *cat* cassette (including Flp recombination site) amplified using Cm1 and Cm2 primers was cloned into the *Pst*I site of pYA4648 to form pYA4649.

### Construction of *Y. pestis* mutant strains

The construction of strains χ10003 and χ10004 using a two-step recombination method was previously described [24]. Strain χ10019 was constructed from strain χ10004 using similar methods. Briefly, plasmid pKD46 was introduced into χ10004 by electroporation. A linear *lacZ-*U-*cat-sacB*-TT *araC* P_BAD_ *spoT*-*lacZ-*D fragment was purified from plasmid pYA4575 by digestion with *Eco*RI and *Sph*I and transformed into χ10004 (pKD46) competent cells. Electroporants were isolated on TBA+Cm (10 µg/ml) plates. Integration of the *lacZ-*U-*cat-sacB*-TT *araC* P_BAD_ *spoT*-*lacZ-*D fragment into the correct site of the chromosome was verified by PCR. Colonies with the correct PCR profile were streaked onto TBA+ Cm (10 µg/ml)+5% Sucrose plates to verify sucrose sensitivity and onto HIB Congo Red+Cm (10 µg/ml) plates to confirm the presence of the *pgm* locus. To remove the *cat-sac* cassette from the chromosome, electrocompetent cells were prepared from a sucrose-sensitive isolate and electroporated with approximately 1 µg of a linear DNA (*lacZ*-U-TT *araC*) cut from pYA4574 using *Sph*I and *Bam*HI. Electroporants were selected on TBA+5% sucrose plates incubated at 30°C. Colonies were tested using PCR to validate that the *cat-sacB* cassette was eliminated. Plasmid pKD46 was cured from a single colony isolate of a sucrose-resistant, chloramphenicol-sensitive strain to yield χ10019.

To construct strains expressing *spoT* tagged with the Flag epitope [25], plasmid pKD46 was introduced into *Y. pestis* KIM6^+^ and χ10019. The resulting strains were electroporated with ∼0.5 µg of *spoTC*-3×*flag*-*kan*-*spoU'* and *spoTC*-3×*flag*-*kan*-*lacZ*-D cut from pYA4645 and pYA4646, respectively. Electroporants were selected on TBA+Kan (20 µg/ml) plates at 37°C. The resulting colonies were verified using PCR to confirm that the 3×*flag*-*kan* fragment was correctly inserted into the chromosome. Plasmid pKD46 was cured from single colony isolates of *Y. pestis* KIM5^+^ or χ10019 derivatives to yield χ10021 and χ10022, respectively.

To construct Pla^−^ mutants, *Y. pestis* KIM6^+^ (pKD46), χ10003 (pKD46), χ10004 (pKD46) and χ10019 (pKD46) competent cells were electroporated with ∼0.5 µg of PCR amplified, gel purified *pla*-U::*cat:pla-*D fragment obtained with primers Pla1 and Pla4 using plasmid pYA4649 as the template. Electroporants were selected on TBA+Cm (10 µg/ml) plates and were subsequently verified by PCR to confirm that *pla* was deleted. Plasmid pCP20 was introduced into the *pla* mutant strains and the Cm^R^ cassette was removed by flip recombinase [26]. Plasmid pCP20 was cured from resulting single colony isolates to yield χ10023, χ10024, χ10025 and χ10026. Then, the pCD1Ap plasmid was transformed into *Y. pestis* KIM6^+^, χ10003, χ10004, χ10019, χ10023, χ10024, χ10025 and χ10026, respectively to form *Y. pestis* KIM5^+^, χ10003(pCD1Ap), χ10004(pCD1Ap), χ10019(pCD1Ap), χ10023(pCD1Ap), χ10024(pCD1Ap), χ10025(pCD1Ap) and χ10026(pCD1Ap) under BSL3 containment.

### ppGpp assay

ppGpp was detected using a slight modification of previously described procedures [27], [28]. To starve cells for amino acids, strains were grown overnight in HIB medium at 26°C. The cells were then harvested and washed three times with PBS and resuspended to an OD_620_ of 0.15 in 1 ml of modified PMH2 medium lacking L-phenylalanine [28]. The culture was shaken at 250 rpm at 26°C for approximately 5 h until the OD_620_ reached 0.25, whereupon, [^32^P] H_3_PO_4_ was added to 100 µCi/ml. Cells were incubated for an additional 1 h at 26°C. Following incubation, an equal amount of chilled 90% formic acid was added to the cell suspension. The ice-cold suspensions were then rigorously vortexed followed by three freeze-thaw cycles. The acid extracts were centrifuged in a minifuge set at the highest speed for 5 min, and 5 µl of supernatant was then applied to a polyethyleneimine–cellulose thin-layer chromatography plate (TLC). The TLC plates were developed at room temperature with 1.5 M KH_2_PO_4_ (pH 3.4). The developed plates were then air-dried and visualized by autoradiography using X-ray film at −70°C. To starve cells for carbon, strains were grown overnight in HIB medium. For strain χ10019, two cultures were grown, one with and one without the addition of 0.05% arabinose. The cells were harvested, washed three times using PBS and resuspended to an OD_620_ of 0.15 in 1 ml of modified PMH2 medium without glucose or arabinose. Cultures were grown, labeled and evaluated by TLC as described above.

### Analysis of virulence factor transcription by RT-PCR

Total RNA was extracted from bacterial cells using TRIzol Reagent (Invitrogen) according to the manufacturer's recommendations. RNA samples were treated with DNase I for 10 min at 37°C to degrade contaminating DNA followed by inactivation of DNase I with 2 mM EDTA and heating to 65°C for 10 min. RNA was then precipitated with sodium acetate and ethanol and washed with 70% ethanol prior to performing RT-PCR. RNA samples of 200 ng were used for reverse transcription, using random hexamer primers and Superscript II reverse transcriptase as described by the manufacturer (Invitrogen). PCR amplification was performed using the *lcrV yopB*, *yopD*, *yopE*, *yopH*, *yopJ*, *yopK*, *yopM*, *yopT* or 16S rRNA primer pairs listed in Supplementary Table S1. RNA samples were used as templates in PCR reactions for RT minus controls. Twenty cycles of amplification were performed using an annealing temperature of 58°C. Products were then separated on a 1% agarose gel, stained with ethidium bromide and imaged for visualization of appropriately sized PCR products. In all cases, reactions were performed in triplicate.

### Protein analysis

Secreted virulence factors were prepared by using a modification of previously described methods [29]. *Y. pestis* was grown in HIB medium overnight at 26°C. The cells were then harvested and washed three times using PMH2, inoculated to 40 ml of fresh PMH2 medium to an OD_600_ of 0.05 and shaken overnight at 26°C. Cultures were shifted to 37°C for 6 h with shaking to provide mild aeration. Bacterial cells were removed by centrifugation for RNA extraction. Secreted virulence factors from the culture supernatants were concentrated by precipitation with 10% (w/v) trichloroacetic acid overnight at 4°C. Precipitated proteins were collected by centrifugation, washed with ice-cold acetone, and dissolved in 0.05 M Tris-HCl buffer (pH 9.5). Insoluble materials were removed by centrifugation at 12 500 *g* for 15 min and the protein concentration in the supernatant was determined using the DC protein assay kit (Bio-Rad Laboratories, Hercules, CA). Samples containing 200 µg proteins were heated at 95°C for 5 min in protein sample buffer containing 2-mercaptoethanol and analyzed by sodium dodecyl sulfate-polyacrylamide gel electrophoresis (SDS-PAGE) with 10% polyacrylamide. Proteins were transferred to nitrocellulose membranes. The membranes were blocked with 5% skim milk in PBS, incubated with rabbit polyclonal antibodies specific for the indicated Yop proteins or LcrV, and washed with PBS-Tween 20. Then alkaline phosphatase-conjugated goat anti-rabbit immunoglobulin G (IgG) (Sigma, St. Louis, MO) was added in PBS-Milk. Immunoreactive bands were detected by the addition of NBT/BCIP (Sigma, St. Louis, MO). The reaction was stopped after 5 min by washing with several large volumes of deionized water.

### Two-dimensional gel electrophoresis

Comparison of two dimensional protein profiles was carried out as previously described [30]. *Y. pestis* KIM5^+^ and χ10004(pCD1Ap) were grown at 26°C or 37°C in 5 ml of best-case-scenario (BCS) medium without Ca^2+^. The cultures were harvested by centrifugation and washed once with low salt PBS (0.1×). Cells were resuspended in 1 ml lysis buffer containing 8M Urea, 0.05M DTT, 2% (w/v) CHAPS and 0.2% (w/v) ampholytes. Proteins were extracted by vortexing 1 ml cell samples in lysis buffer with 0.2 mm glass beads ten times for 30 s with cooling between vortexing. The samples were centrifuged at 2500 *g* for 5 min to remove the beads. The bead-free supernatant was centrifuged at 15000 *g* for 15 min at 4°C to remove cellular debris. The cell-free lysates were immediately placed on ice and protease inhibitor was added. The lysates were retreated with a 2D protein cleanup kit (Bio-Rad, Hercules, CA) and protein concentration was determined using the Bio-Rad Protein Assay kit.

Protein lysates (300 µg) were mixed with rehydration buffer (Bio-Rad) in a total volume of 300 µL. Equal amounts (300 µg) of protein were isoelectrically focused using 17 cm pH 4–7 strips followed by 18.3×19.3 cm 8–16% SDS–PAGE using Midi-Protean II 2D cell (Bio-Rad). Gels were stained with Coomassie Brilliant Blue R-250 (Bio-Rad) and visualized using Gel Doc XR system (Bio-Rad). Protein expression levels from protein spots on gels were compared between the different samples. Gel analysis was performed using the PDQuest3 2-D Analysis Software (Bio-Rad) to determine differential expression. Differentially expressed protein spots were excised and were digested with In-Gel Tryptic Digestion Kit (Pierce, Rockford, IL). Peptide digests were analyzed using a Voyager DE STR MALDI-TOF mass spectrometer (Applied Biosystems, Framingham, MA). Data were searched in bacterial proteomics database using Aldente in ExPASy Proteomics Server. This experiment was performed four times with similar results.

### Virulence studies in mice

Single colonies of each strain were used to inoculate HIB cultures and grown overnight at 26°C. To select for plasmid pCD1Ap, ampicillin was added into the medium at a concentration of 25 µg/ml. Bacteria were diluted into 10 ml of fresh HIB enriched with 0.2% xylose and 2.5 mM CaCl_2_ to obtain an OD_620_ of 0.1 and incubated at 26°C for s.c. infections (bubonic plague) or at 37°C for intranasal (i.n.) infections (pneumonic plague). Both cultures were grown to an OD_620_ of 0.6. The cells were then harvested and the pellet resuspended in 1 ml of isotonic PBS.

All animal procedures were approved by the Arizona State University Animal Care and Use Committee. Female 7-week-old Swiss Webster mice from Charles River Laboratories were inoculated by s.c. injection with 100 µl of bacterial suspension. Actual numbers of colony-forming units (CFU) inoculated were determined by plating serial dilutions onto TBA agar. To determine 50% lethal dose (LD_50_), five groups of six mice were infected with serial dilutions of the bacterial suspension. For in vivo complementation of strain of χ10019(pCD1Ap), 120 mg of L-arabinose dissolved in PBS was intraperitoneally administered to mice on the day of inoculation and once a day thereafter [31]. Mice were monitored twice daily for 21 days, and the LD_50_ was calculated as described [32].

For colonization/dissemination analysis, 3 mice per time point were infected by s.c. injection in the front of the neck. At the indicated times after infection, mice were euthanized, and samples of blood, lungs, spleen and liver were removed. The bacterial load for each organ was determined by plating dilutions of the homogenized tissues onto TBA with ampicillin plates and reported as CFU per gram of tissue or CFU per ml blood. Infections were repeated in at least two independent experiments.

### Preparation of bacterial antigens

Bacterial antigens used for ELISA were prepared from fresh cells. Briefly, single colonies of *Y. pestis* KIM5^+^ were inoculated into HIB media and cultured overnight at 26°C. Cells were switched to 37°C for 6 h. Bacterial cultures were centrifuged at 5,000×*g* for 10 min, the pellet was washed once with sterile PBS and resuspended in sterile PBS. Bacterial cells were broken using 0.2 mm glass beads 10 times for 60 s with cooling between vortexing (with 2 min incubation on ice between cycles). The whole bacterial lysate was sterilized by UV light and sterility was confirmed by TBA agar culture. The lysate was frozen at −80°C until use. Protein content was determined by BCA analysis per manufacturer's instructions (Sigma).

### Enzyme-linked Immunosorbent Assay (ELISA)

Mice were lightly anesthetized using ketamine and xylazine mixture administered intramuscularly. Blood was collected by retro-orbital sinus puncture for the determination of antibody titers at different time points. ELISA was used to assay serum antibodies against the whole cell lysate of *Y. pestis* KIM5^+^. Sera were tested for IgG at a starting dilution of 1∶1000, and for IgG1 and IgG2a at 1∶100, respectively.

Polystyrene 96-well flat-bottom microtiter plates (Dynatech Laboratories Inc., Chantilly, VA) were coated with 200 ng/well of *Y. pestis* whole cell lysates. Antigens suspended in sodium carbonate-bicarbonate coating buffer (pH 9.6) were applied in 100 µl volumes to each well. The coated plates were incubated overnight at 4°C. Free binding sites were blocked with a blocking buffer (phosphate-buffered saline [PBS; pH 7.4], 0.1% Tween 20, and 1% bovine serum albumin). A 100 µl volume of serially diluted sample was added to individual wells in triplicate and incubated for 1 h at 37°C. Plates were treated with biotinylated goat anti-mouse IgG, IgG1, or IgG2a (Southern Biotechnology Inc., Birmingham, AL). Wells were developed with streptavidin-horseradish peroxidase conjugate (Invitrogen, Carlsbad, CA), followed by 2,2-azino-bis-(3-ethylbenzthiazoline-6-sulfonic acid) (ABTS) (Sigma) in sodium citrate buffer containing 0.03% hydrogen peroxide (H_2_O_2_). After a 10 min incubation at 37°C in the dark, color development (absorbance) was recorded at 405 nm using an automated ELISA plate reader (model EL311SX; Biotek, Winooski, VT). Absorbance readings that were 0.1 higher than PBS control values were considered positive.

### In vivo cytokine analysis

Cytokines were quantitated by a double-sandwich enzyme-linked immunosorbent assay (ELISA) as described previously [33]. Mice in groups of three were euthanized at intervals by terminal bleeding under anesthesia. Pooled blood was allowed to clot overnight at 4°C, and serum was separated by centrifugation at 10,000 g for 10 min. Sera were filtered once through a 0.22 µm syringe filter, cultured on TBA to confirm that bacteria had been removed and stored at −70°C prior to assay for cytokines.

Commercial solid-phase enzyme immunoassays utilizing the multiple-antibody sandwich principal were used to determine cytokines in biological samples. In these experiments, IL-10, TNF-α and IFN-γ were determined with Mouse IL-10, IFN-γ and TNF-α Ready-SET-Go kits (ebioscience), respectively. Concentrations of cytokines were measured by reading optical density at 450 nm and then calculated in reference to values obtained in standard curves generated for each assay. Assays of pooled sera were repeated three times.

### Protective efficacy

Two groups of Swiss Webster mice (10 mice/group) were immunized by s.c. injection with 2.5×10^4^ CFU of χ10004(pCD1Ap) cells in 100 µl of isotopic PBS on day 0. Two groups of mice (4 mice/group) were injected with 100 µl of PBS as controls. On day 35, animals were challenged by s.c. injection with 100 µl of virulent *Y. pestis* KIM5^+^ or lightly anesthetized with a 1∶5 xylazine/ketamine mixture and challenged by the intranasal route with 20 µl of bacterial suspension. The challenge dose for s.c. injection was 1×10^5^ CFU and for i.n. challenge was 2.0×10^4^ CFU. Protective efficacy was determined by the number of surviving animals. All infected animals were observed over a 15-day period for the development of signs of plague infection.

### Statistical analysis

Data are expressed as means±SE. One-way analysis of variance with Student t-test were used for statistical analysis. A *P*-value of<0.05 was considered significant.

## Results

### Sequence analysis of the *relA* and *spoT* genes

Analysis of the *Y. pestis* KIM5^+^ database revealed the presence of *relA* and *spoT* genes homologous to *E. coli* K-12 and *S*. Typhimurium LT-2 [34], [35], [36]. The *Y. pestis* RelA protein shares 84.7% identity with *E. coli* K-12 and 83.9% identity with *S*. Typhimurium LT-2 RelA proteins. The *Y. pestis* SpoT protein has 91.3% identity with *E. coli* K-12 and 91.8% identity with *S*. Typhimurium LT-2 SpoT proteins.

Our analysis indicated that *Y. pestis* SpoT, but not RelA, possesses the HD domain that is conserved in a superfamily of metal-dependent phosphohydrolases [37]. Histidine (H) and aspartate (D) residues in the HD domain are thought to be involved in (p)ppGpp degradation [37]. Both *Y. pestis* RelA and SpoT proteins possess the conserved ATP/GTP-binding and GTP binding domains, TGS [38] and ACT [39], [40], respectively, that are present in the *E. coli* RelA and SpoT proteins [41]. The presence of these conserved motifs in the *Y. pestis* proteins is in agreement with their biochemical functions because ATP and GTP are substrates of the reaction catalyzed by (p)ppGpp synthetase.

### The *relA* and *spoT* genes are involved in synthesis of ppGpp and physiological differentiation

**Figure 1:**
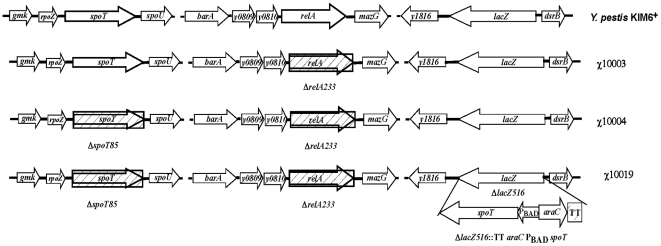
Schematic chromosome structure of *Y. pestis* KIM6^+^, χ10003 (Δ*relA233*), χ10004 (Δ*relA233*Δ*spoT85*) and χ10019 (Δ*relA233* Δ*spoT85* Δ*lacZ*::TT *araC* P_BAD_ *spoT*).

To evaluate the linkage between *relA* and *spoT* and the production of ppGpp, we constructed *ΔrelA*, *ΔrelA ΔspoT* [24] and *ΔrelA ΔspoT* Δ*lacZ*::TT *araC* P_BAD_ *spoT* mutants of *Y. pestis* KIM6^+^ strain (Fig. 1). To construct a strain with arabinose-regulated *spoT* expression, a TT *araC* P_BAD_ promoter cassette was inserted in front of the *spoT* gene. The *spoT* gene is located in the middle of an operon. To avoid affecting the transcription of nearby genes, the TT *araC* P_BAD_ *spoT* construct was inserted at another location, *lacZ* (Fig. 1).

**Figure 2:**
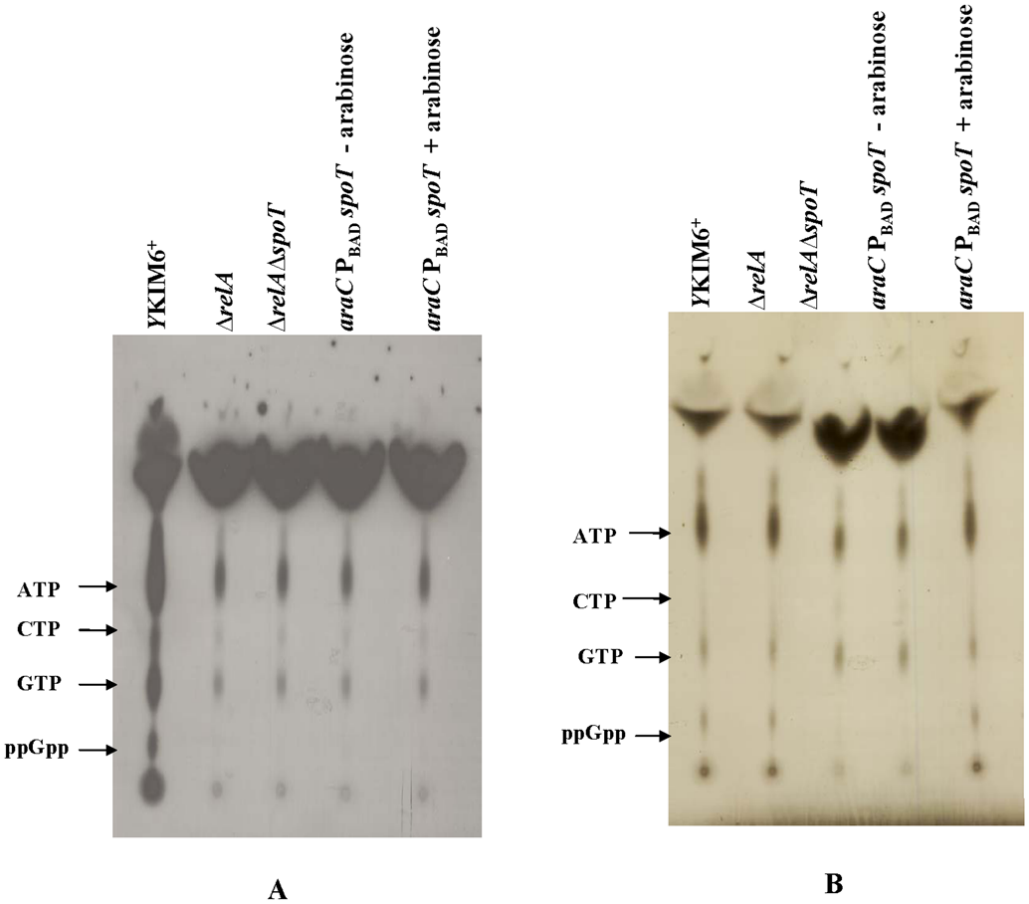
Analysis of (p)ppGpp synthesis in *Y. pestis* KIM6^+^ and Δ*relA ΔspoT* mutants during amino acid and carbon starvation by TLC. Total intracellular nucleotides were extracted from *Y. pestis* cultures uniformly labeled with [^32^P] H_3_PO_4_. Cells were grown in modified PMH2 medium lacking L-phenylalanine for amino acid starvation (A) and in modified PMH2 medium without glucose for carbon starvation (B).

Because of the high degree of similarity between *Y. pestis* RelA and SpoT proteins and their *E. coli* and *Salmonella* counterparts, it is likely that the function of RelA and SpoT in *Y. pestis* will be the same. To evaluate the effect of *relA* and *spoT* on ppGpp synthesis during amino acid starvation, *Y. pestis* was grown in PHM2 media [21] without L-phenylalanine. ppGpp accumulation was observed in wild-type *Y. pestis*, but not in the *relA* null strains (Figure 2A), illustrating that *Y. pestis* is indeed capable of ppGpp biosynthesis in response to amino acid starvation. We also evaluated the effect of carbon starvation. When glucose was exhausted in the medium, ppGpp accumulated in the wild type and *ΔrelA spoT^+^* strains, but not in *ΔrelA ΔspoT* strains (Figure 2B). These results indicate that *Y. pestis* has a RelA-dependent response to amino acid starvation and a SpoT-dependent response to glucose starvation, comparable to what is observed in *E. coli* [42]. The SpoT deficiency could be complemented in strain χ10019 (Δ*relA233* Δ*spoT85* Δ*lacZ516*::TT *araC* P_BAD_ *spoT*) by the addition of arabinose. Synthesis of SpoT in strain χ10019 in the presence of 0.05% arabinose was nearly identical to wild-type SpoT synthesis (Fig. S1 and S2). The addition of arabinose to strain χ10019 also restored ppGpp synthesis when cells were starved for carbon (Fig. 2B).

**Figure 3:**
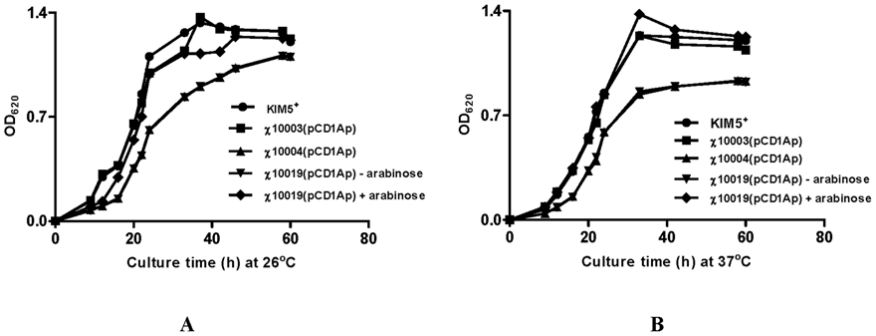
Growth of *Y. pestis* strains in HIB medium at different temperatures (A) Growth curve at 26°C; (B) Growth curve at 37°C. •, *Y. pestis* KIM5^+^; ▪, χ10003(pCD1Ap) (Δ*relA233*) ▴, χ10004(pCD1Ap) (Δ*relA233*Δ*spoT85*); ▾, χ10019(pCD1Ap) (Δ*relA233* Δ*spoT85* Δ*lacZ*::TT *araC* P_BAD_ *spoT*) without arabinose; ♦, χ10019(pCD1Ap) (Δ*relA233* Δ*spoT85* Δ*lacZ*::TT *araC* P_BAD_ *spoT*) with 0.05% arabinose.

A cursory examination of the *Y. pestis* Δ*relA* Δ*spoT* double mutant after growth on solid rich medium indicated that the Δ*relA* Δ*spoT* double mutants grew more slowly than wild-type or Δ*relA* mutants. When growth was assessed in liquid medium, the Δ*relA* Δ*spoT* mutants exhibited a longer lag phase and did not reach as high a final OD_600_ than the wild-type and Δ*relA* mutant strains at both 26°C and 37°C (Fig. 3A and B). The Δ*relA* Δ*spoT* strains were prone to autoaggregate and precipitate to the bottom of the culture tube at 26°C, but not at 37°C (data not shown). The addition of 0.05% arabinose restored wild-type growth characteristics to strain χ10019 (Δ*relA233* Δ*spoT85* Δ*lacZ516*::TT *araC* P_BAD_ *spoT*) (Fig. 3), but it continued to autoaggregate and precipitate at 26°C. However, the addition of higher concentrations of arabinose reduced autoaggregation in a concentration-dependent manner. The addition of 0.4% arabinose resulted in the complete absence of detectable autoaggregation at 26°C.

### The effect of ppGpp on production of virulence factors of *Y. pestis*

**Figure 4:**
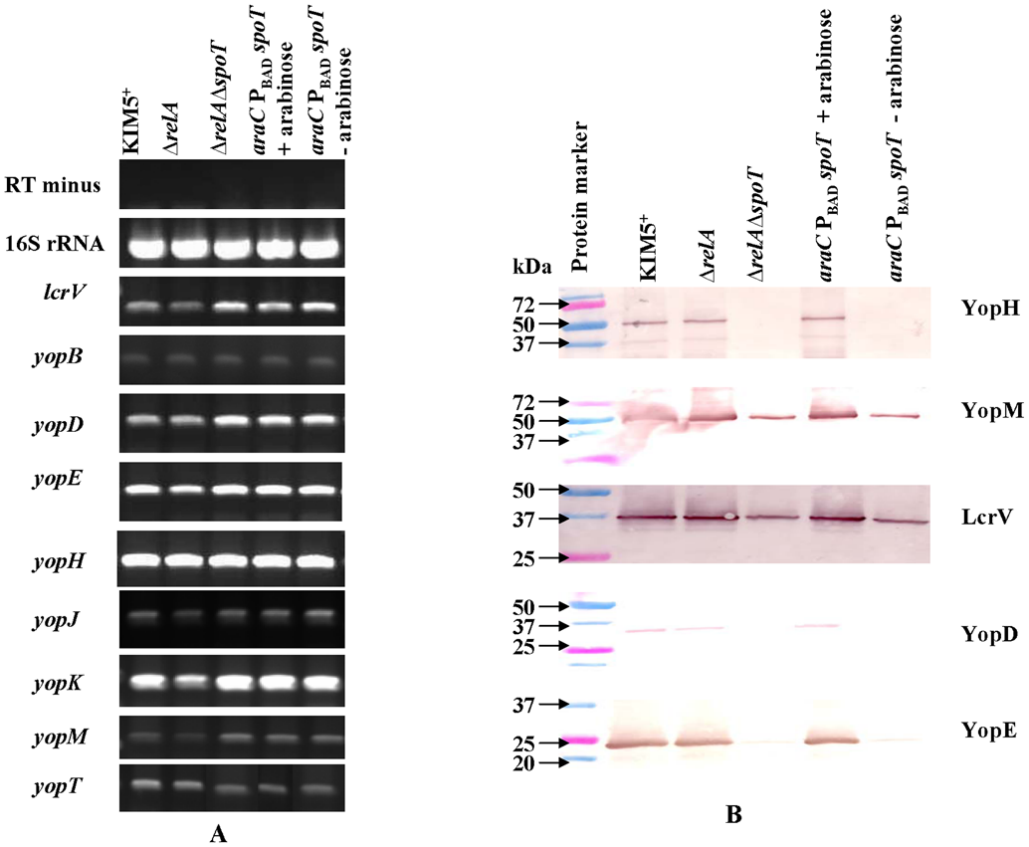
Analysis of virulence factor expression and secretion in *Y. pestis* KIM5^+^ and mutants. (A) Evaluation of virulence factor transcription by semi-quantitative RT-PCR. (B) Measurement of secreted virulence factors in culture supernatants by western blotting. Secreted proteins were collected from the culture medium following the removal of bacterial cells. Proteins were separated by SDS-PAGE and detected by western blotting. For each sample, the same amount of total protein was loaded.

The virulence of the pathogenic *Yersinia* species depends on a plasmid-encoded type III secretion system (T3SS) that transfers effector proteins called Yops (*Yersinia* outer proteins) into host cells, interfering with mammalian cell signaling pathways, inhibiting phagocytosis, modulating cytokine production, and inducing apoptosis [43]. In *S.* Typhimurium, pathogenicity islands 1 and 2 (SPI1 and SPI2) encode T3SSs required for invasion and replication within host cells, respectively [44]. SPI1 and SPI2 gene transcription and expression are severely reduced in the absence of ppGpp [45]. To determine if ppGpp had a similar effect on *Y. pestis*, transcription of the genes encoding T3SS substrates LcrV and Yop proteins was analyzed using RT-PCR. Our results indicated that *relA* or *relA spoT* status did not have a significant effect on the transcription of *lcrV* and or the *yop* genes (Fig. 4A).

To examine the effect of ppGpp on protein synthesis, the proteome of wild-type and *ΔrelA ΔspoT* mutant *Y. pestis* strains was compared at different temperatures using two-dimensional electrophoresis (Fig. S3). Our results indicate that deletion of *relA* and *spoT* led to reduced synthesis of some metabolic enzymes at flea (26°C) and human (37°C) temperatures, and also reduced synthesis of virulence factors such as Pla, LcrH and LcrV at 37°C (Table 2 and Table 3).

**Table 2:** Differentially expressed proteins identified from *Y. pestis* at 26°C.

Protein number	Protein name	Accession No.	Function	Method	Fold change
					WT/Δ*relA*Δ*spoT*
1	PanC (pantoate–beta-alanine ligase)	y0785	biosynthesis of cofactors, carriers: pantothenate	MALDI	7.3
2	hypothetical protein	y2262	putative	MALDI	15.2
3	S-ribosylhomocysteinase	y0888	catalyzes the hydrolysis of S-ribosylhomocysteine to homocysteine and autoinducer-2	MALDI	8.6
4	MetG (methionyl-tRNA synthetase)	y2648	aminoacyl tRNA synthetases, tRNA modification	MALDI	2.7
5	PyrE (orotate phosphoribosyltransferase)	y0096	pyrimidine ribonucleotide biosynthesis	MALDI	2.5
6	PyrB (aspartate carbamoyltransferase catalytic Subunit)	y0161	pyrimidine ribonucleotide biosynthesis	MALDI	3.6

**Table 3:** Differentially expressed proteins identified from *Y. pestis* at 37°C.

Protein number	Protein name	Accession No.	Function	Method	Fold change
					WT/Δ*relA*Δ*spoT*
1	LcrH (SycD) secretion chaperone	YPCD1.30c	chaperone for YopBD	MALDI	2.3
2	FrsA (fermentation/respiration switch protein)	y0964	FrsA may promote fermentation	MALDI	2.8
3	MetK (S-adenosylmethionine synthetase)	y3314	catalyzes the formation of S-adenosylmethionine from methionine and ATP; methionine adenosyltransferase	MALDI	4.2
4	CodA (cytosine deaminase)	y3946	salvage of nucleosides and nucleotides	MALDI	1.5
5	Pla (outer membrane protease)	YPPCP1.07	outer membrane protease; involved in virulence in many organisms	MALDI	2.6
6,7,8	LcrV (secreted effector protein)	YPCD1.31c	functions in needle complex protein export; Yop secretion and targeting control protein; important for translocation pore formation	MALDI	7.3
9	TrpA (tryptophan synthase subunit alpha)	y2047	amino acid biosynthesis: Tryptophan	MALDI	1.6
10	TyrS (tyrosyl-tRNA synthetase)	y1966	aminoacyl tRNA synthetases, tRNA modification	MALDI	1.6
11	hypothetical protein	y2786	putative membrane protein	MALDI	2.3
12	Kbl (2-amino-3-ketobutyrate coenzyme A ligase)	y0081	Central intermediary metabolism: pool, multipurpose conversions	MALDI	1.7

We also evaluated secretion of LcrV and some of the Yops. Recovery of secreted Yop proteins is hampered by degradation due to Pla activity [46]. Therefore, secretion of virulence factors was evaluated in Δ*pla* derivatives, χ10023(pCD1Ap) (Δ*pla*), χ10024(pCD1Ap) (Δ*pla* Δ*relA*), χ10025(pCD1Ap) (Δ*pla ΔrelA ΔspoT*) and χ10026(pCD1Ap) (Δ*pla* Δ*relA* Δ*spoT araC* P_BAD_ *spoT*). The results indicate that LcrV and YopM secretion was reduced slightly in absence of ppGpp (*ΔrelA ΔspoT*), but secretion of YopH, YopD and YopE were significantly decreased (Fig. 4B).

### A Δ*relA ΔspoT* mutant is attenuated in mice

**Figure 5:**
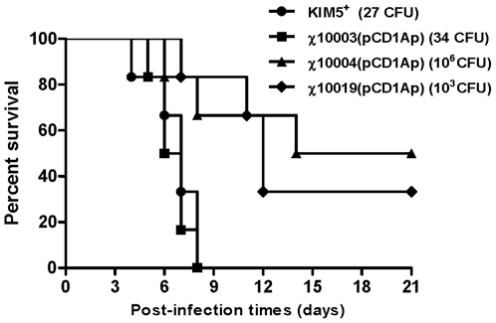
Survival of Swiss Webster mice (3 mice per strain) infected s.c. with *Y. pestis* KIM5^+^ (black circles), χ10003(pCD1Ap) (black squares), χ10004(pCD1Ap) (black triangles) and χ10019(pCD1Ap) cultured with 0.05% arabinose *in vitro* (black diamonds). The experiment was performed twice with similar results.

To investigate the contribution of ppGpp to the virulence of *Y. pestis*, we infected groups of three Swiss Webster mice subcutaneously with wild-type, χ10003(pCD1Ap) (Δ*relA233*), χ10004(pCD1Ap) (*ΔrelA233 ΔspoT85*) and χ10019(pCD1Ap) (Δ*relA233* Δ*spoT85* Δ*lacZ516*::TT *araC* P_BAD_ *spoT*), in which *spoT* expression is regulated by arabinose availability. Strain χ10019(pCD1Ap) was grown in the presence of arabinose prior to inoculation of mice. Once this strain colonizes host tissues where there is no free arabinose [23], it will become phenotypically SpoT^−^. In preliminary experiments we determined that the LD_50_ of the wild-type strain in mice is<10 CFU, consistent with previous results [47], [48]. Mice given wild-type *Y. pestis* KIM5^+^ and χ10003(pCD1Ap) (Δ*relA*) succumbed to the infection in a highly synchronous manner (Fig. 5). Only 50% of the mice infected with 5.8×10^5^ CFU of *ΔrelA ΔspoT* strain χ10004 developed plague after 6 days, and the rate at which the mice died was slower than the rate of those infected with the wild-type strain. The LD_50_ of χ10004(pCD1Ap) was 5.8×10^5^ CFU. Thus, the lack of ppGpp resulted in a ∼100,000-fold increase in the LD_50_ obtained by subcutaneous (s.c.) infection. The LD_50_ of χ10019(pCD1Ap) strain, administered after growth in arabinose was intermediate, at 3.3×10^2^ CFU (∼100-fold increase). The LD_50_ of χ10019(pCD1Ap) was the same as KIM5^+^ (LD_50_<10) when inoculated mice were injected with arabinose, indicating full complementation of the attenuation phenotype.

**Figure 6:**
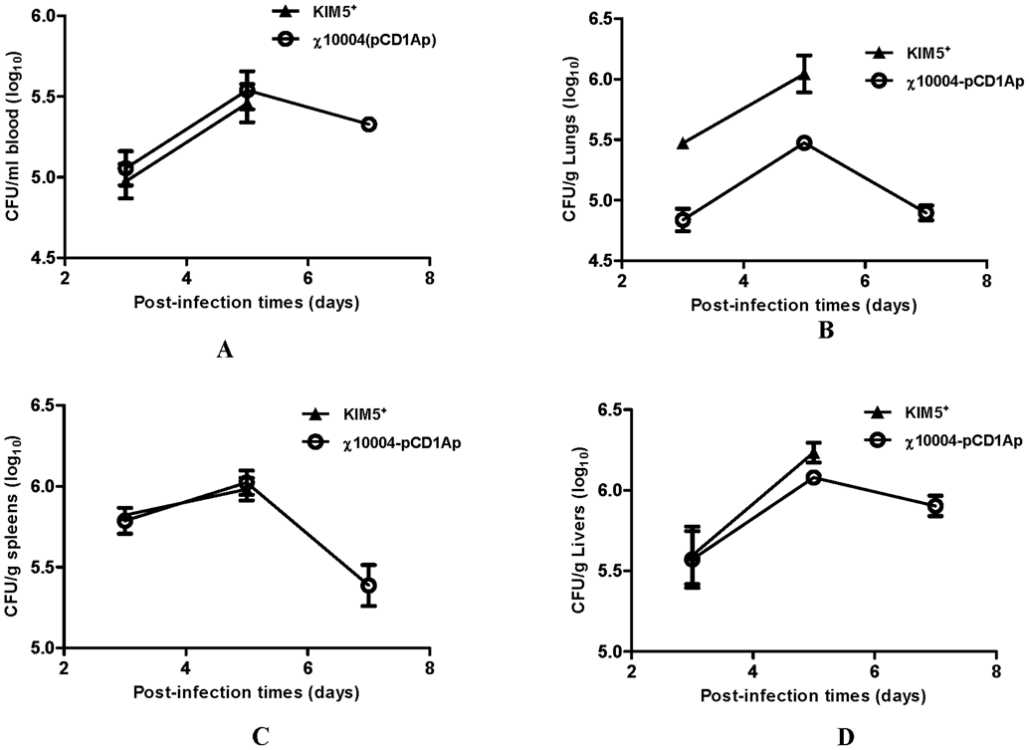
Kinetics of infection with *Y. pestis* KIM5^+^ (black) or χ10004(pCD1Ap) (white) in mouse tissues. Groups of nine mice were inoculated s.c., and at various times CFU per organ in the blood (A), lungs (B), spleens (C) and livers (D) were determined for 3 mice per group. Error bars represent standard deviation.

To further evaluate the ability of *Y. pestis* to disseminate to the bloodstream and internal organs, we monitored the growth of both *Y. pestis* KIM5^+^ and χ10004(pCD1Ap) in the lungs, spleens, livers and blood of infected mice over a 7-day period after s.c. injection. Because of the difference in LD_50_ between the two strains, we inoculated mice with different doses of each, 1.5×10^3^ CFU of *Y. pestis* KIM5^+^ or 1.6×10^6^ CFU of χ10004(pCD1Ap). The kinetics of colonization was similar for both strains (Fig. 6). Despite the difference in dose, the levels of bacteria in blood, spleen and liver were similar for both strains on days 3 and 5. There was an approximate 1.5 log difference in bacteria isolated from lung tissue, indicating that the *ΔrelA ΔspoT* mutant had was less efficient than KIM5^+^ at reaching the lungs. By day 7, the number of the *ΔrelA ΔspoT* mutant began to decline in all tissues, indicating clearance by the host, while all of the mice inoculated with wild-type *Y. pestis* had succumbed to the infection.

### The immune responses to Δ*relA* Δ*spoT Y. pestis* strain χ10004(pCD1Ap)

**Figure 7:**
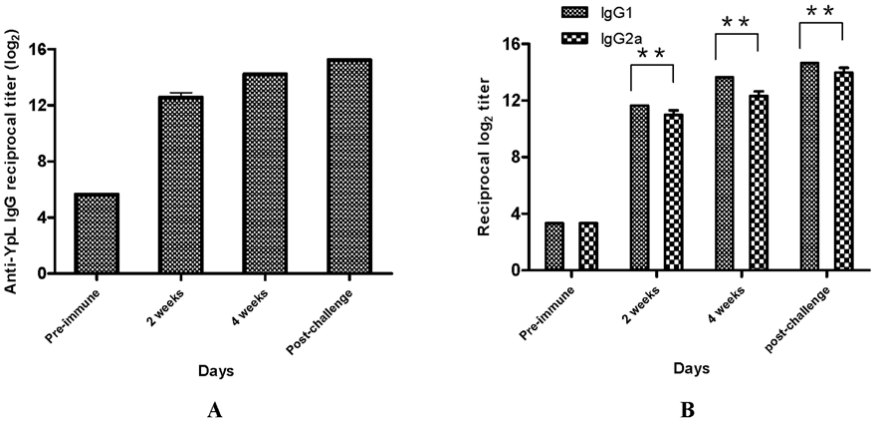
Antibody response in sera of mice inoculated with *Y. pestis* KIM5^+^ or χ10004(pCD1Ap). A *Y. pestis* whole cell lysate was used as the coating antigen. (A) Serum IgG responses. (B) Serum IgG1 and IgG2a responses. *, the *P* value was less than 0.01; **, the *P* value was less than 0.05.

Because χ10004 was attenuated, we explored its potential as a vaccine. To evaluate the immune responses to *ΔrelA ΔspoT Y. pestis* strain χ10004(pCD1Ap), two groups of 10 mice each were immunized s.c. with 2.5×10^4^ CFU on day 0. Two groups of 4 mice each were injected with PBS as controls. Mice were challenged on day 35 with either 1.5×10^5^ (s.c.) or 2.0×10^4^ (i.n.) CFU of *Y. pestis* KIM5^+^. Blood was taken at 2 and 4 weeks post immunization and 2 weeks after challenge. Serum IgG responses to *Y. pestis* whole cell lysates (YpL) from immunized mice were measured by ELISA (Fig. 7A). At two weeks after immunization, the reciprocal anti-*Y. pestis* serum IgG titers were greater than 1,000 and increased at 4 weeks and after challenge.

The serum immune responses to YpL were further examined by measuring the levels of IgG isotype subclasses IgG1 and IgG2a. Th1 cells direct cell-mediated immunity and promote class switching to IgG2a, and Th2 cells provide potent help for B-cell antibody production and promote class switching to IgG1 [49]. The level of anti-YpL IgG1 and IgG2a isotype antibodies rapidly increased after vaccination and gradually increased at 2 weeks, 4 weeks and post-challenge (Fig. 7B). At 2 and 4 weeks post-immunization, the ratio of IgG1 to IgG2a was 1.06∶1 and 1.2∶1 respectively, indicating an initial mixed Th1/Th2 response, which developed into a slight Th2 bias by week 4. This Th2 bias continued after challenge as well.

### Immunization with a Δ*relA* Δ*spoT Y. pestis* strain χ10004(pCD1Ap) can protect against plague challenge

**Figure 8:**
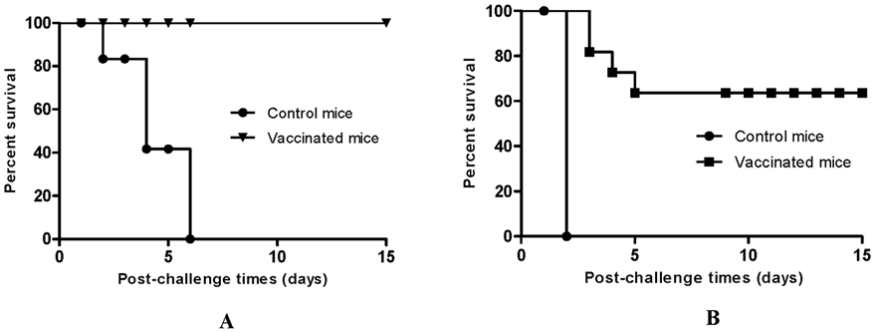
Mouse survival after *Y. pestis* KIM5^+^ Challenge. (A) Swiss Webster mice vaccinated s.c. with 2.5×10^4^ CFU of χ10004(pCD1Ap) and a were challenged with 1.5×10^5^ CFU of *Y. pestis* KIM5^+^ via the s.c. route. (B) Swiss Webster mice vaccinated s.c. with 2.5×10^4^ CFU of χ10004(pCD1Ap) were challenged via the i.n. route with 2×10^4^ CFU of *Y. pestis* KIM5^+^. Immunization provided significant protection against both challenge routes (*P*<0.001). For each experiment, there were 10 mice in the vaccinated group and 4 mice in the control group.

To evaluate the protective efficacy of *ΔrelA ΔspoT Y. pestis* strain χ10004(pCD1Ap) against the bubonic and pneumonic forms of plague, immunized mice were challenged on day 35 with either 1.5×10^5^ (s.c.) or 2.0×10^4^ (i.n.) CFU of *Y. pestis* KIM5^+^. Post-challenge survival was monitored for 14 days. A single s.c. vaccination could provide complete protection against s.c. challenge without any symptoms (Fig. 8A) and 60% protection against pulmonary challenge (Fig. 8B). None of the mice immunized with PBS survived either challenge (Fig. 8).

### Induction of cytokines by *Y. pestis* KIM5^+^ and *ΔrelA ΔspoT* strain χ10004(pCD1Ap)

**Figure 9:**
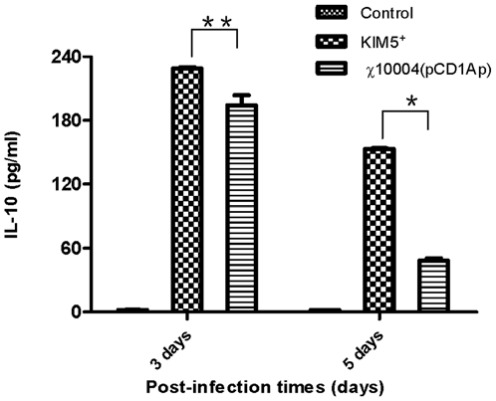
IL-10 production in sera of mice inoculated with *Y. pestis* KIM5^+^ or χ10004(pCD1Ap). *, the P value was less than 0.01; **, the *P* value was less than 0.05.

Cytokines are critical to the development and functioning of both the innate and adaptive immune responses. They are often secreted by immune cells that have encountered pathogens, thereby activating and recruiting additional immune cells to increase the system's response to the pathogen. Previously, LcrV has been demonstrated to be an immunomodulator (TNF-α and IFN-γ down-regulation and IL-10 induction) both *in vivo* and *in vitro* [50], [51], [52]. Since the synthesis and secretion of LcrV is reduced in the *ΔrelA ΔspoT* mutant, we compared production of three cytokines (IL-10, INF-γ and TNF-α) in mice infected with *Y. pestis* KIM5^+^ and χ10004(pCD1Ap). For this experiment, groups of three Swiss-Webster mice were inoculated via the s.c. route with 1.5×10^3^ CFU of *Y. pestis* KIM5^+^ or 1.6×10^6^ CFU of χ10004(pCD1Ap). A group of uninfected mice served as controls. Blood was collected via cardiac puncture 3 and 5 days later for cytokine analysis. Measurements indicated that levels of IL-10 were higher in the sera of animals infected with *Y. pestis* KIM5^+^ than that of χ10004(pCD1Ap) (Fig. 9). The pro-inflammatory cytokines IFN-γ and TNF-α were not detected in sera from mice inoculated with either strain (data not shown).

## Discussion

The bacterial global signal molecule, ppGpp, plays a major role in translating the nutritional state of bacteria into appropriate expression of virulence genes. Our studies indicated that *relA* and *spoT* deletion mutants of *Y. pestis* and *S.* Typhimurium have similar, but not identical, effects on growth and virulence. A *ΔrelA* deletion does not affect the growth of either *Y. pestis* (Fig. 3) or *S.* Typhimurium (data not shown). In addition, as is the case for *S.* Typhimurium, the *Y. pestis ΔrelA ΔspoT* mutants fail to reach the same final cell density as their wild-type parents (Fig. 3) [18]. However, the *ΔrelA ΔspoT Y. pestis* strain showed a slight growth defect in vitro, a phenomenon not reported for *S.* Typhimurium [18].

The *ΔrelA ΔspoT* mutant was prone to autoaggregate and precipitate at 26°C. This phenotype was not apparent at 37°C. This phenomenon was never reported in *ΔrelA ΔspoT* mutants of *Salmonella* and *E. coli*. However, we could find no published experiments where the mutants had been grown at 26°C or a similar low temperature. To investigate whether this phenotype could be observed in *S.* Typhimurium, we cultured wild type, Δ*relA*, and Δ*relA* Δ*spoT S.* Typhimurium UK-1 at 26°C and 37°C. We did not observe autoaggregation or precipitation at 26°C (data not shown), indicating that this phenotype is specific for *Y. pestis*. In addition, autoaggregation was not alleviated in strain χ10019 (Δ*relA* Δ*spoT araC* P_BAD_ *spoT*) by addition of 0.05% arabinose, a concentration that allowed complementation of other phenotypes relating to ppGpp formation (Fig. 2), growth (Fig. 3) and Yop and LcrV secretion (Fig. 4B). Autoaggregation was, however, eliminated by the addition of 0.4% arabinose, indicating that a higher level of *spoT* expression was required to alleviate this phenotype.

ppGpp regulates expression of many genes involved in the virulence and pathogenesis of *S.* Typhimurium including SPI-1 and SPI-2 encoded genes essential for invasion and the *spv* operon, involved in systemic infections [44], [45]. In *Y. pestis*, the 70-kb virulence plasmid, pCD1, encodes a complex virulence property called the low-Ca^2+^ response (LCR) [53], [54]. The LCR was first observed in vitro, where the bacteria respond to the absence of Ca^2+^ at 37°C by the strong expression and secretion of Yops and LcrV [4]. This is accompanied by growth restriction, in which the yersiniae undergo an orderly metabolic shutdown and cease growth [55], [56]. The phenomenon is phenotypically similar to the stringent response which is induced by ppGpp synthesized when cells encounter amino acid or carbon starvation [10]. However, early research indicated that *Y. pestis*, although capable of ppGpp biosynthesis, did not utilize this pathway to mediate its temperature-dependent response to Ca^2+^-deficient environments. Early lesions in RNA synthesis associated with the growth restriction of Ca^2+^-deprived *Y. pestis* reflected a block in stable RNA synthesis and this effect is not mediated by ppGpp [28]. Yops accumulate in the membrane fraction after 3 h of induction, whereas transcription of the *yop* genes during Ca^2+^-deficiency is dramatically reduced [57]. Our results showed that the *ΔrelA ΔspoT* mutations did not affect *yop* transcription, but resulted in reduced synthesis and secretion of LcrV and a number of Yops, including YopD. YopD is essential for several discrete steps during efficient Yop effector translocation [58], therefore, the reduction of YopD synthesis might directly impair the secretion of other Yops.

A number of studies report the involvement of ppGpp in processes related to growth, stress, starvation, and survival that affect pathogenicity. A frequent scenario is that in *relA spoT* mutants, pathogenicity is compromised for reasons that vary with the organism studied and include pathogen/host interactions, invasiveness and persistence [59]. Our observations indicate that the *relA*-dependent accumulation of ppGpp does not play a major role in *Y. pestis* pathogenicity. The *ΔrelA Y. pestis* mutant was as virulent as wild type *Y. pestis* KIM5^+^ (Fig. 6), while the Δ*relA* Δ*spoT Y. pestis* mutant was dramatically attenuated. The growth defect observed in vitro for the *ΔrelA ΔspoT* strain may have contributed to this reduction in virulence. Zusman *et al* showed that an *L. pneumophila relA* mutant is capable of wild-type intracellular proliferation in both human macrophage and the protozoan host *Acanthamoeba castellanii* [15]. A *S.* Typhimurium Δ*relA* mutant also showed no significant differences in invasion, intracellular growth, virulence, or expression of a number of SPI-1 genes [18]. Unlike *S.* Typhimurium, wild-type *Y. pestis* and *L. pneumophila* are not capable of growth on defined medium unless supplemented with amino acids that also serve as a carbon source for these pathogens [60], [61], [62]. The pattern of amino acid requirements of *Y. pestis* is similar to that of the *Legionella* [60] and *S.* Typhimurium Δ*relA* Δ*spoT* strains [63], including two branched chain amino acids, phenylalanine and threonine. But amino acid auxotrophs designed to duplicate the amino acid requirements of Δ*relA* Δ*spoT* mutants do not affect intracellular growth and virulence of *Legionella* [64], [65] and *Salmonella* [18]. Therefore, we conclude that it is the basal (SpoT-dependent) ppGpp levels that affect pathogenicity of *Y. pestis* and not the stringent response induced by amino acid starvation.

The increased LD_50_ and lung colonization deficiency of the ppGpp null mutant indicates that ppGpp may regulate genes important in establishing a lethal infection during bubonic plague. Only a modest degree of attenuation was observed in the pneumonic model (unpublished data). The pneumonic model bypasses the need to reach the lungs, indicating that it is the ability to reach the lungs, and not the ability to colonize the lungs, per se, that is at least partially responsible for the attenuation phenotype. This suggests that *relA* and *spoT* are required for expression of genes that facilitate lung invasion after subcutaneous entry into the host. Furthermore, this observation highlights the point that mutant strains are not universally attenuated for virulence, and route of infection is an important consideration when investigating the phenotype of a mutant.

Results of proteome analysis indicated that the absence of ppGpp in *Y. pestis* reduced Pla and LcrH synthesis at 37°C (Table 3). This may be another important factor leading to attenuation. Pla is a member of the omptin surface protease family and cleaves host plasminogen and components of the complement pathway [66]. Independent of this protease activity, Pla binds to the extracellular matrix component laminin and promotes invasion of endothelial cells [67]. Inactivation of *pla* severely attenuates *Y. pestis* during bubonic infection [68], [69]; however, a *pla* mutant is still lethal during intranasal or intravascular infection [70]. In addition, the YopE and YopH type III effector proteins disrupt the host cell cytoskeleton allowing the bacterium to resist phagocytosis [71], [72] and are required for *Y. pestis* virulence [20], [73]. Therefore, the reduction in YopE and YopH synthesis observed in the Δ*relA* Δ*spoT* mutant may also contribute to attenuation.

High titers of anti-*Y. pestis* serum IgG were produced by vaccination with the Δ*relA* Δ*spoT Y. pestis* mutant and antibody-based humoral immunity provides good protection against bubonic plague [74], [75]. Although a mixed Th1/Th2-type immune response was initially induced by this attenuated strain, the response became slightly biased to a Th2-type humoral immune response. *Y. pestis* secretes LcrV and Yops during infection. LcrV triggers the release of IL-10 by host immune cells and suppresses proinflammatory cytokines such as TNF-α and INF-γ as well as innate defense mechanisms required to combat the pathogenesis of plague [76], [77]. Our results were similar to previous findings. We were unable to detect TNF-α and IFN-γ in sera of mice inoculated with the wild-type or the ppGpp null mutant, but the IL-10 levels induced by the ppGpp null mutant were significantly lower than that of wild type strain (Fig. 7). The reduction in LcrV expression in the ppGpp null mutant could be responsible for the decrease in IL-10 production. However, the reduced expression of LcrV and Yops was not sufficient to permit induction of the proinflammatory cytokines TNF-α and IFN-γ.

Subcutaneous immunization with Δ*relA* Δ*spoT* strain χ10004(pCD1Ap) protected mice against s.c. challenge (simulating bubonic plague) and i.n. challenge (simulating pneumonic plague), but protection against i.n. challenge was not complete (Fig. 8) in spite of high serum titers of anti-*Y. pestis* IgG (Fig. 7). This result is consistent with what is known about the immunogenicity requirements for protection against *Y. pestis*. Protection against bubonic plague is primarily antibody-mediated [74], [75], while protection against pneumonic plague may require cell mediated immunity induced by INF-γ and TNF-α [78], [79]. This view is supported by vaccine trials with nonhuman primates indicating that humoral immunity may not protect humans against pulmonary *Y. pestis* infection. Specifically, studies by researchers at the USAMRIID found that a significant number of nonhuman primates immunized with the F1-V fusion protein vaccine succumbed to aerosol *Y. pestis* challenge, despite high-titer serum antibodies against F1-V at the time of challenge [80], [81], [82]. Therefore, antibodies alone are not sufficient to protect against pneumonic plague. Recent studies demonstrated that cytokine-mediated immunity could augment protection against lethal pulmonary *Y. pestis* challenge in the presence or absence of specific antibody [79], [83]. Therefore, absence of TNF-α and IFN-γ in sera may be the major reason we observed poor protection efficacy against pneumonic plague.

Based on these results, an effective plague vaccine needs to prime not only humoral immunity but also strong Th1-type cellular immunity [79], [84]. To induce effective cellular immunity we will further modify our Δ*relA* Δ*spoT* strain to permit induction of cellular immune responses.

## Supporting Information

Table S1 Primers
(0.07 MB DOC)

Figure S1 Schematic chromosome structure of *Y. pestis* KIM6+, χ10021 (*spoT412*:: 3×flag-kan), χ10019 (Δ*relA233* Δ*spoT85* Δ*lacZ516*::TT *araC* PBAD *spoT*) and χ10022 (Δ*relA233* Δ*spoT85* Δ*lacZ516* ΩTT *araC* PBAD *spoT413*:: 3×flag-kan).
(0.23 MB TIF)

Figure S2 Measurement of SpoT expression M, protein marker; 1, *Y. pestis* KIM6+; 2, χ10021; 3, χ10022 (without arabinose); 4, χ10022 (with 0.05% arabinose); 5, χ10022 (with 0.1% arabinose); 6, χ10022 (with 0.15% arabinose); 7, χ10022 (with 0.2% arabinose); 8, χ10022 (with 0.3% arabinose).
(0.90 MB TIF)

Figure S3 2-DE gels showing differential protein expression A. Comparing differential protein expression between KIM5+(wild-type *Y. pestis*) and χ10004-pCD1Ap (Δ*relA233* Δ*spoT85*) at 26°C. B. Comparing differential protein expression between KIM5+(wild-type *Y. pestis*) and χ10004-pCD1Ap (Δ*relA233* Δ*spoT85*) at 37°C.
(1.16 MB TIF)
