# Supplementary material for: The Role of relA and spoT in Yersinia pestis KIM5+ Pathogenicity
Source: PLoS One. 2009 Aug 24;4(8):e6720. doi: 10.1371/journal.pone.0006720 (PMC2726946; doi:10.1371/journal.pone.0006720)
Supplement: Table S1 — Primers (0.07 MB DOC) [file pone.0006720.s001.doc]

TABLE S 1. Oligonucleotides used in this work

| Name | Sequence |
| --- | --- |
| LacZ1 ^a^ | 5’ cggctgcagcccatcactccagcgcagaact 3’ (*Pst*I) |
| LacZ2 | 5’ cgggcatgctccagcccattcaggcttat 3’(*Sph*I) |
| LacZ3 | 5’ cgggaattccaaaggagcaatgcatgtatgg 3’ (*Eco*RI) |
| LacZ4 | 5’ cgggagctccatgtgttgccaactggctg 3’ (*Sac*I) |
| LacZ5 | 5’ ctaaattgttatctcttcgtag 3’ |
| LacZ6 | 5’ tgcagggagatgagttaacaatg 3’ |
| SpoT-1 ^a, b^ | 5’ cggctcgag**GGAGTG**aaacg**ttg**tacctgtttgaaagcct 3’ (*Xho*I) |
| SpoT-2 ^a^ | 5’ cgggagctcttaattgcgattacggctaactttaacc3’ (*Sac*I) |
| Pla1 | 5’ cgggaattcagcaaaacagacaaacgcctgctgg 3’ (*Eco*RI) |
| Pla2 | 5’ cggctgcagtagacacccttaatctctctgcatg 3’ (*Pst*I) |
| Pla3 | 5’ cggctgcagtacagatcatatctctcttttcatcctc 3’ (*Pst*I) |
| Pla4 | 5’ cgggcatgcctggtgcgtatagctgaggatgaat 3’ (*Sph*I) |
| Pla5 | 5’ gagataacgtgagcaaaacaaaatctggtcg 3’ |
| Pla6 | 5’ gagccttttatgcgttcgatccgattcg 3’ |
| Cm1 | 5’cggaactgcagatgggaattagccatggtcc 3’ (*Pst*I) |
| Cm2 | 5’cggctgcagtgtaggctggagctgcttcg 3’ (*Pst*I) |
| SpoTC-1 | 5’ cggaagcttatgagcgtagtggtggctaa 3’ (*Hind*III) |
| SpoTC-2 | 5’ cggggatccattgcgattacggctaactt 3’ (*Bam*HI) |
| SpoTD-1 | 5’cgggagctctaacgcctatgaatcctcaacgctatg 3’ (*Sac*I) |
| SpoTD-2 | 5’ cgggaattctgtgtgtccgtttatacatc 3’ (*Eco*RI) |
| Flag-1 | **5’ cggggatccgactacaaagaccatgacggtgatt 3’ (*Bam*HI)** |
| Flag-2 | **5’ cgggagctccatatgaatatcctccttagttcctat 3’ (*Sac*I)** |
| Cm-V | 5’gttgtccatattggccacgttta3’ |
| SacB-V | 5’ gcagaagagatatttttaattgtggacg 3’ |
| araC-V | 5’catccaccgatggataatcgggta3’ |
| 16S rRNA primer1 | 5’ aggcgacgatccctagctggtctga 3’ |
| 16S rRNA primer2 | 5’ cgtttacagcgtggactaccagggt 3’ |
| lcrV primer1 | 5’ tcctagcttattttctacccgagga 3’ |
| lcrV primer2 | 5’ ttaattcggcggtaagctcagctaa 3’ |
| yopB primer1 | 5’ tgtttcagtgctaacgaagtttacgc 3’ |
| yopB primer2 | 5’ acaatcactgaggctatggcgctga 3’ |
| yopD primer1 | 5’ tcttgttgttgctgttggaactggc 3’ |
| yopD primer2 | 5’ gttgttcgcggccagcaatattact 3’ |
| yopE primer1 | 5’ catttgctgcctgcgttagatcaac 3’ |
| yopE primer2 | 5’ gccaaaatacatgcagcagttgaat 3’ |
| yopH primer1 | 5’ tcgtcaggtatctcgattggtgcag 3’ |
| yopH primer2 | 5’ ccattgccgacacttcttaagtcat 3’ |
| yopJ primer1 | 5’ tcacgtatggatgtagaagtcatgc 3’ |
| yopJ primer2 | 5’ gtttttgtccttattgccagcatcg 3’ |
| yopK primer1 | 5’ gtgctttatgtaccgctcttgaaca 3’ |
| yopK primer2 | 5’ gtcaatatcgctgacatgttgccat 3’ |
| yopM primer1 | 5’ acgtcattcttctaatttaactgagatg 3’ |
| yopM primer2 | 5’ aagtgatttcaggctctgcggtaat 3’ |
| yopT primer1 | 5’ tcaaggatagcgtttaataattgatccag 3’ |
| yopT primer2 | 5’ tttatgtgcacattggatcaggagc 3’ |

* a: the restriction endonuclease sites are underlined

b: the bold capital letters show the Shine-Dalgarno (SD) sequence and the TTG start codon
