# Supplementary figures and images for: The Role of relA and spoT in Yersinia pestis KIM5+ Pathogenicity
Source: PLoS One. 2009 Aug 24;4(8):e6720. doi: 10.1371/journal.pone.0006720 (PMC2726946; doi:10.1371/journal.pone.0006720)

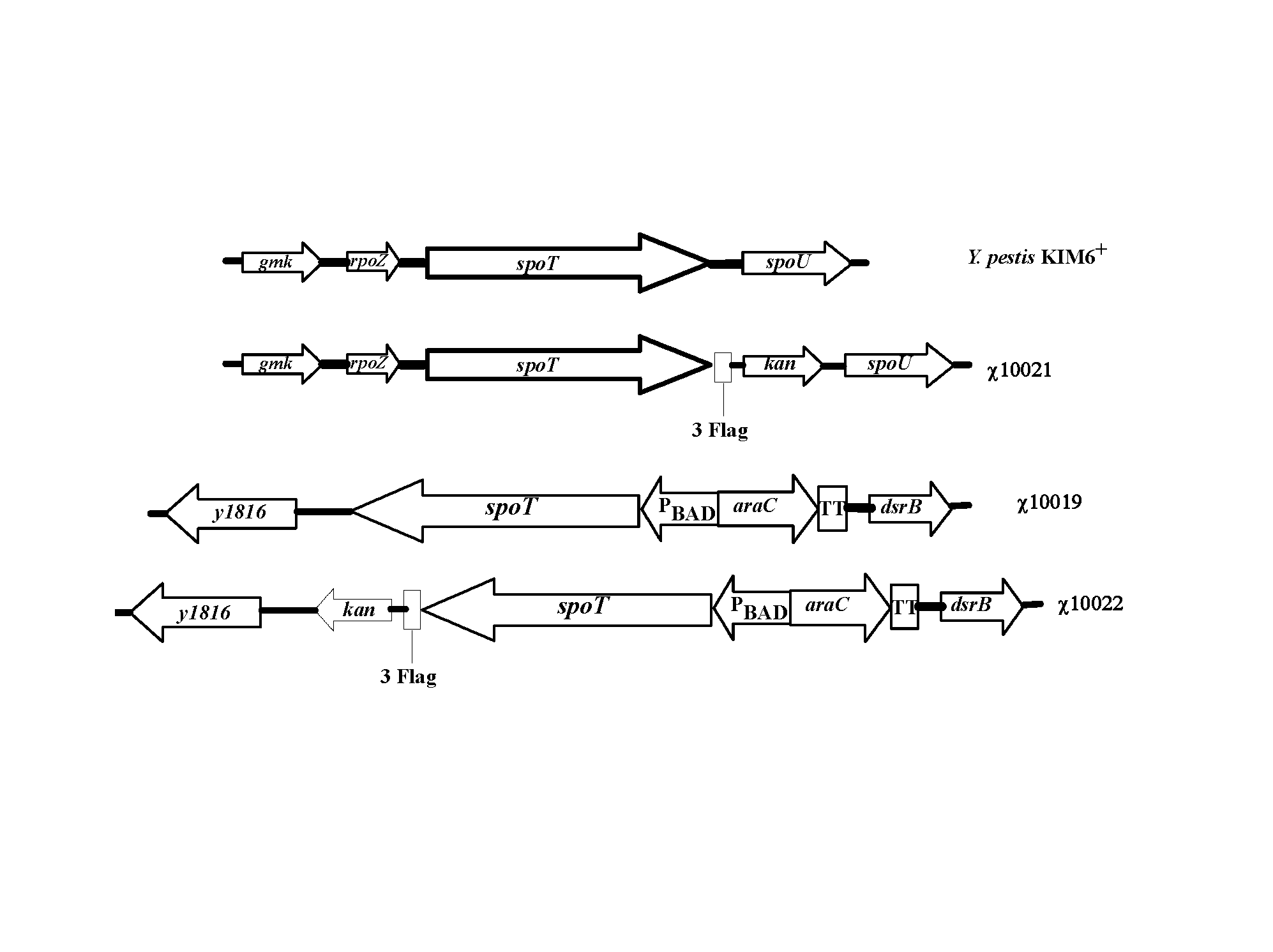

Supplement: Figure S1 — Schematic chromosome structure of Y. pestis KIM6+, χ10021 (spoT412:: 3×flag-kan), χ10019 (ΔrelA233 ΔspoT85 ΔlacZ516::TT araC PBAD spoT) and χ10022 (ΔrelA233 ΔspoT85 ΔlacZ516 ΩTT araC PBAD spoT413:: 3×flag-kan). (0.23 MB TIF) [file pone.0006720.s002.tif]

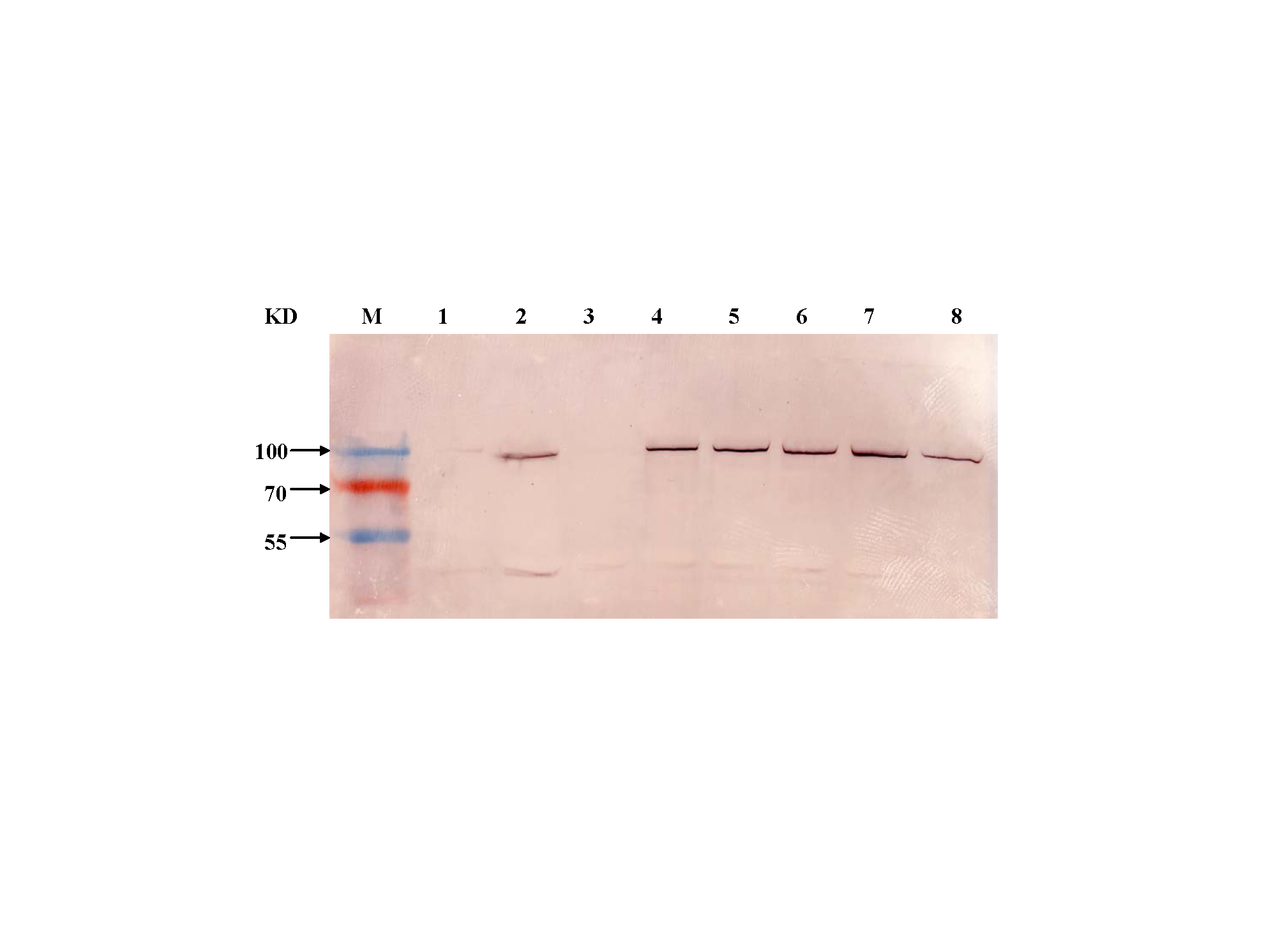

Supplement: Figure S2 — Measurement of SpoT expression M, protein marker; 1, Y. pestis KIM6+; 2, χ10021; 3, χ10022 (without arabinose); 4, χ10022 (with 0.05% arabinose); 5, χ10022 (with 0.1% arabinose); 6, χ10022 (with 0.15% arabinose); 7, χ10022 (with 0.2% arabinose); 8, χ10022 (with 0.3% arabinose). (0.90 MB TIF) [file pone.0006720.s003.tif]

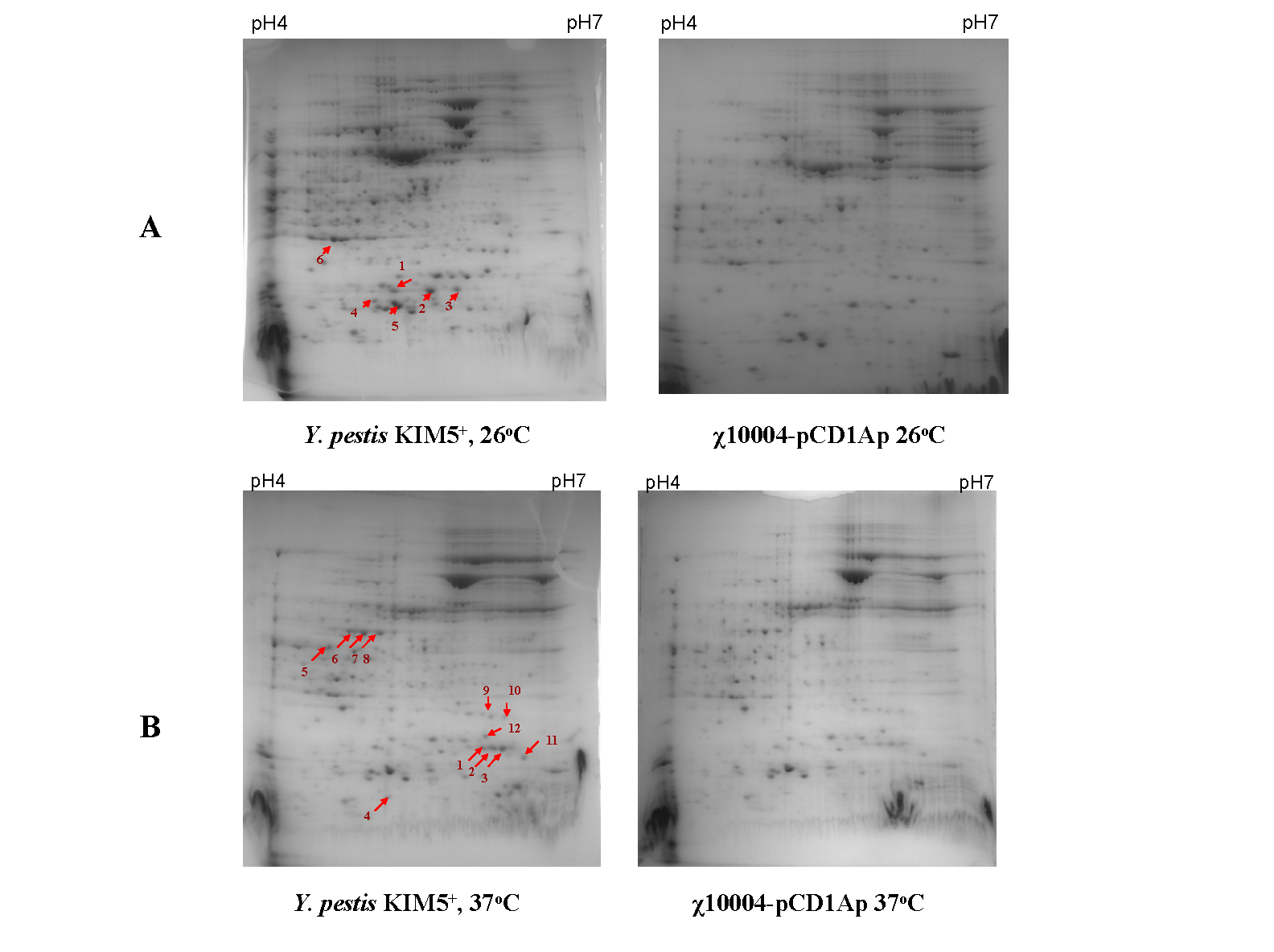

Supplement: Figure S3 — 2-DE gels showing differential protein expression A. Comparing differential protein expression between KIM5+(wild-type Y. pestis) and χ10004-pCD1Ap (ΔrelA233 ΔspoT85) at 26°C. B. Comparing differential protein expression between KIM5+(wild-type Y. pestis) and χ10004-pCD1Ap (ΔrelA233 ΔspoT85) at 37°C. (1.16 MB TIF) [file pone.0006720.s004.tif]
